# Mendelian randomization study supports effect of gut microflora on fractures

**DOI:** 10.1097/MD.0000000000037017

**Published:** 2023-02-02

**Authors:** Ling-Ling Ju, Yong-Kang Wei, Yanjun Liu

**Affiliations:** a Institute of Biomedical Engineering, College of Medicine, Southwest Jiaotong University, Chengdu, Sichuan, China; b The Fourth Clinical Medical College of Xinjiang Medical University, Urumqi, Xinjiang, China

**Keywords:** fracture, gut, Mendelian randomization, microflora, risk

## Abstract

To investigate the possible causal relationship between intestinal microflora and fractures using Mendelian randomization (MR). A 2-sample MR study of gut microbiota and fractures was conducted using a weighted inverse variance analysis with tests for heterogeneity, horizontal pleiotropy, and sensitivity. A causal association between fracture risk and specific bacterial taxa was identified at various taxonomic levels: 2 (*Bacteroidia, P* = .0304; *Deltaproteobacteria P* = .0304) at the class level, 3 (*Bacteroidales, P* = .0428; *Desulfovibrionales, P* = .0428; *Enterobacteriales, P* = .0208) at the order level, 2 (FamilyXI, *P* = .0304; *Enterobacteriaceae P* = .0332) at the family level, and 1 (*Alistipes, P* = .0405) at the genus level. This study revealed a causal relationship between gut microflora and fracture risk, demonstrating that the effect of different flora taxa flora abundance on fracture risk differs. It provides a reference for further studies.

## 1. Introduction

The human gut is inhabited by thousands of bacteria that affect host physiology through unique mechanisms of action, and these bacterial taxa are collectively referred to as the gut microbiota.^[1]^ Microbiologists and clinicians have increasingly emphasized the clinical value of intestinal microflora in the last decade, and several studies have been conducted to identify the association of intestinal microflora with various human diseases and abnormal physiological mechanisms. For example, the effect of gut microflora on obesity,^[2]^ diabetes,^[3]^ human aging,^[4]^ psychiatric disorders,^[5]^ and cardiovascular diseases, etc.^[6]^ The relationship between the gut microflora and the human body has opened a new chapter.

A fracture, as we all know, is a disruption of the continuity and stability of the bone. It is 1 of the most common diseases in orthopedics and is associated with various factors, mainly external trauma, aging, and bone loss.^[7]^ Recent studies have revealed the association between gut microflora and bone and have further explored the relationship between gut microflora and bone health.^[8]^ An observational study found a possible correlation between gut microflora and fracture risk.^[9]^ The gut microbiota regulates osteoporosis through the “brain-gut-bone” axis, and Guo et al showed that *Lactobacillus rhamnosus* GG can improve osteoporosis by regulating the T helper cell 17/Treg balance. Osteoporosis is a major risk factor for bone fractures, which may indicate that our gut microflora may play an important role in the occurrence of bone fractures.^[10,11]^ However, no other evidence supports an association between gut microflora and fracture risk.

Mendelian randomization (MR) is a reliable epidemiological method for proving causality using information about genetic variation as an instrumental variable.^[12]^ Its strong evidence-based medical evidence could better demonstrate the association between gut microflora and fracture risk. Genome-wide association studies (GWAS) have allowed us to explore the relationship between gut microbiota and fracture risk further.

## 2. Materials and methods

### 2.1. Study design

This study used 2-sample MR to investigate the possible causal relationship between gut microbiota and fracture risk. Approximately 196 gut microbial taxa with unique species names recorded in MiBioGen were used as exposures. About 10 Fracture information from FinnGen includes forearm fractures, lower leg fractures including ankle, shoulder and upper arm fractures, lumbar spine and pelvis fractures, wrist and hand fractures, foot fractures (except ankle), femur fractures, rib, sternum, thoracic spine fractures, neck fractures, and skull and facial bone fractures were used to represent fracture outcomes.

### 2.2. Data sources

Gut microbiota data were obtained from MiBioGen (https://mibiogen.gcc.rug.nl/), which included 340,024 individuals from 18 cohorts, most of European ancestry (*n* = 13,266), examined 211 gut microbiota abundances, and recorded a total of 122,110 associated single nucleotide polymorphisms (SNPs) (15 unnamed taxa were excluded from this study.). All 10 outcome variables related to fractures were obtained from the FinnGen Biobank (Round 9) (https://r9.finngen.fi/), a large GWAS study based on the Finnish biobank Project, with sample sizes and information on nSNPs shown in the table below (Table 1). The diagnostic classifications of fractures were based on Iinternational Classification of diseases-10, and the present study defined the phenotypes (Table 1).

**Table 1:** Outcome information.

Outcome	nCases	nContrils	Samplesize	nSNP
Fracture of forearm	19,577	351,196	370,773	20,170,116
Fracture of lower leg, including ankle	19,994	323,307	343,301	20,169,406
Fracture of shoulder and upper arm	11,750	345,173	356,923	20,169,771
Fracture of lumbar spine and pelvis	6209	364,504	370,713	20,170,079
Fracture at wrist and hand level	11,394	336,418	347,812	20,169,593
Fracture of foot, except ankle	7593	351,392	358,985	20,169,888
Fracture of femur	8766	360,928	369,694	20,170,071
Fracture of rib(s), sternum and thoracic spine	9094	362,260	371,354	20,170,108
Fracture of neck	1503	370,193	371,696	20,170,126
Fracture of skull and facial bones	6907	329,318	336,225	20,169,238

The data are all from the European population but from different countries, so the outcomes are relatively independent of the exposed GWAS data, and there is no significant sample overlap.

The data used in this study were obtained from publicly available GWAS, with links provided for inclusion in the text, and therefore did not require ethical review.

### 2.3. Filtering of instrumental variables

We used *P* = 1e^−5^ as a threshold to screen relevant SNPs as possible instrumental variables (IV), thus ensuring a solid correlation of IV. To remove the interference between SNPs due to linkage disequilibrium, clump analysis was performed, and populations with nSNPs < 3 were excluded, thus ensuring independence between the final IV SNPs. Finally, the F-statistic was calculated with the formula F = beta2/se2; the SNPs with F-statistic < 10 were excluded, and reliable SNPs were obtained as IV.^[13]^

### 2.4. Data analysis

All statistical analyses and plots were performed in Rstudio software based on R4.3.1, and the main R package used was “TwosampleMR.”

A 2-sample MR method was used, with inverse variance weighted as the primary method and MR-Egger and Weighted Median as supplements for MR analysis. Cochrane’s Q test, leave-one-out test, and MR-Egger intercept test were used to perform heterogeneity tests, sensitivity analyses, and tests of horizontal multiple validity. When there is horizontal multiplicity, the analytical discussion is meaningless. When there is no horizontal pleiotropy and no heterogeneity, a fixed effects model is used to represent and judge the results. When there is no horizontal pleiotropy and heterogeneity, the random-effects model represents and judges the results.^[14]^ To reduce the occurrence of type II error, the *P* value of the inverse variance weighted (IVW) results were corrected hierarchically from the phylum-family-genus-species level of the intestinal microflora using the Benjamini–Hochberg method.^[15]^

The results screened for the absence of pleiotropy, and the corrected *P* value was still less than the 0.05 threshold, which was used as the result. The heterogeneity of the results was again checked using the MR-PRESSO Global test, and depending on the heterogeneity of the results, either a fixed-effects model or a random-effects model was selected to represent the final result. The positive results were summarized and presented.

## 3. Results

**Figure 1.**
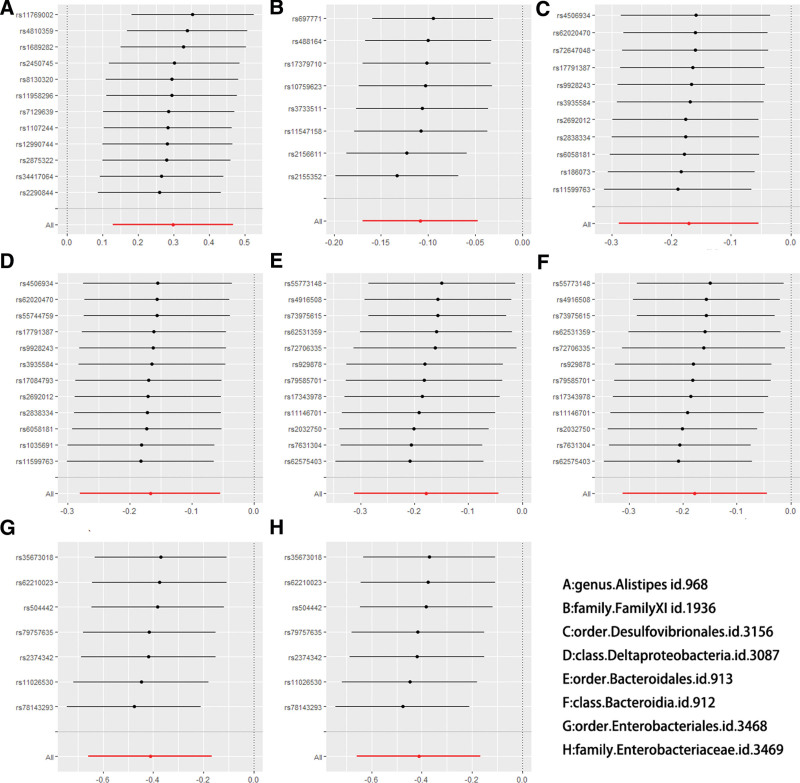
Sensitivity analysis of positive results.

**Figure 2.**
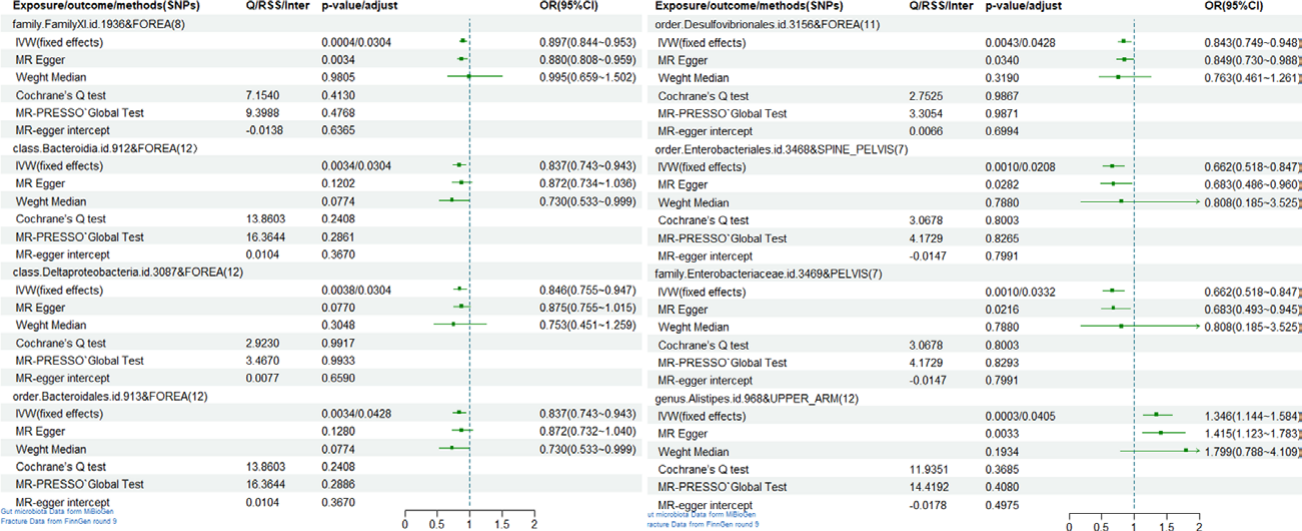
Forest plot of positive results of Mendelian randomization analysis.

Excluding results with horizontal pleiotropy and selecting the type of IVW method used based on heterogeneity, we finally found 2 taxa of bacteria, *Bacteroidia* and *Deltaproteobacteria*, associated with the risk of fracture occurrence at the class level and 3 taxa of bacteria, *Bacteroidales, Desulfovibrionales*, and *Enterobacteriales*, were associated with fracture risk at the order level, *Desulfovibrionales* and *Enterobacteriales* were associated with fracture risk at the order level, 2 taxa of bacteria, FamilyXI and *Enterobacteriaceae*, were associated with fracture risk at the family level, and *Alistipes* was associated with fracture risk at the genus level (Figs. 1 and 2).

**Figure 3.**
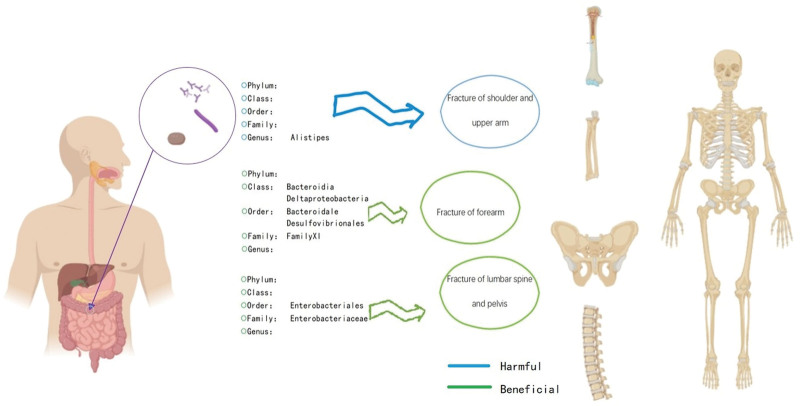
Schematic representation of the effect of gut microflora on fracture risk at different sites.

It was tested that none of the above positive results were heterogeneous with horizontal pleiotropy, so the fixed effects model was chosen to explain the results. After performing the leave-one-out test, the results were stable and reliable (Figs. 2 and 3).

In MR analysis at the Class level, we see that *Bacteroidia* [IVW (fixed effects): P-adjust = 0.0304, OR (95%CI) = 0.837 (0.743~0.943)], *Deltaproteobacteria* [IVW (fixed effects): P-adjust = 0.0304, OR (95%CI) = 0.846 (0.755~0.947)], both taxa of *Bacteroidia* were causally associated with Fracture of forearm, with a 16.3% decrease in the risk of Fracture of forearm occurring with each 1 standard deviation increase in the abundance of *Bacteroides*. The risk of Fracture of the forearm decreased by 15.4% with each standard deviation increase in the abundance of *Deltaproteobacteria*.

In the order level MR analysis, we see that *Bacteroidales* [IVW (fixed effects): P-adjust = 0.0428, OR (95%CI) = 0.837 (0.743~0.943)], *Desulfovibrionales* [IVW (fixed effects): P-adjust = 0.0428, OR (95%CI) = 0.843 (0.749~0. 948)] were causally associated with a forearm fracture, *Enterobacteriales* [IVW (fixed effects): P-adjust = 0.0208, OR (95%CI) = 0.662 (0.518~0.847)] were causally associated with a lumbar spine fracture, and pelvis was causally associated. The results suggest that with each standard deviation increase in the abundance of *Bacteroidia*’s flora, we have a 16.3% decrease in the risk of occurrence of fracture of the forearm. For each standard deviation increase in the abundance of *Desulfovibrionales*, the risk of forearm fracture decreased by 15.7%. For each standard deviation increase in the abundance of *Enterobacteriales*, the forearm fracture risk was reduced by 33.8%.

In the MR analysis at the family level, we see that FamilyXI [IVW (fixed effects): P-adjust = 0.0304, OR (95%CI) = 0.897 (0.844~0.953)] was causally associated with forearm fracture and *Enterobacteriaceae* [IVW (fixed effects): P-adjust = 0.0332, OR (95%CI) = 0.662 (0.518~0.847)] was causally related to lumbar and pelvic fracture. The results suggest that we decreased the risk of forearm fracture occurrence by 103% with each standard deviation increase in the abundance of the flora of FamilyXI. Lumbar spine and pelvis fracture risk decreased by 33.8% with each standard deviation increase in the population abundance of *Enterobacteriaceae*.

At the genus level, we found only a causal association between *Alistipes* [IVW (fixed effects): P-adjust = 0.0405, OR (95% CI) = 1.346 (1.144–1.584)] and fracture of the shoulder and upper arm. The results suggest that the shoulder and upper arm fracture risk increased by 34.6% with each standard deviation increase in the abundance of *Alistipes* flora.

Detailed analysis results, data used, code, specific IV, and other images that may be required can be obtained by contacting the author at ningq14@foxmail.com.

## 4. Discussion

Based on the published GWAS study, this study performed a 2-sample MR analysis on 10 exposures of selected gut microbiota and surrogate fractures. The results showed that (1) there was a causal relationship between gut microbiota and fracture. (2) The effect of gut microbiota on fracture was mainly protective but also a risk factor. (3) The abundance of the genus *Alistipes* flora was a risk factor for fracture. (4) class *Bacteroidia*, class *Deltaproteobacteria*, order *Bacteroidales*, order *Desulfovibrionales*, order *Enterobacteriale*, family *Enterobacteriaceae* flora abundance as a protective factor for fracture (Fig. 3).

Previous studies have also examined gastrointestinal microflora and fracture risk. For example, a cohort study from Japan involving 38 postmenopausal women found an increased fracture risk in a cohort with a lower abundance of the genus *Bacteroidales*, corroborating our findings.^[9]^

The exact mechanism of the effect of gut microflora on fracture risk is unclear. The impact on bone mineral density (BMD) may influence fracture risk through its effect on BMD. Many studies have reported the impact of gut microflora on BMD, suggesting that our gut microflora may increase the risk of fragility fractures by affecting inflammatory cytokines that lead to bone loss through nutrient absorption.^[16]^

Gut microflora can affect BMD from an endocrine perspective and an immune response perspective through the “microbe-gut-bone” axis. Evidence suggests intestinal microflora can affect the secretion of 5-hydroxytryptamine, insulin-like growth factor 1, and parathyroid hormone, thus affecting BMD.^[17–19]^ Gut microflora can also influence the regulation of immune-inflammatory cells such as T helper cell 17, CD4-positive T-lymphocytes, and Treg cells, activating the corresponding pathways and ultimately causing a chronic inflammatory response that affects bone mineral density and fracture risk.^[8,20,21]^ Gut microorganisms can also influence critical miRNAs in osteoblasts’ transcriptional process, thus affecting osteogenesis’s operation, such as the effect on miRNA-33-5p, which may affect the differentiation of osteoblasts.^[22]^

Observational studies have also shown that the abundance of *Bacteroides* and *Roseburia* in the intestinal microflora of osteoporotic patients is often reduced, as suggested by studies of the intestinal microflora of osteoporotic patients.^[23,24]^ The present research indicates that a decrease in the abundance of *Bacteroidales* may increase fractures, confirming that gut microflora may influence fracture risk through a pathway that affects bone mineral density.

The effect of gut microflora on related vitamins may also be associated with fracture risk. Members of the gut microbiota are known to synthesize vitamin K and most water-soluble B vitamins, including biotin, cobalamin, folic acid, niacin, pantothenic acid, pyridoxine, riboflavin, and thiamin.^[25]^ For example, vitamin K is a potential protective component against fractures, and the gut microflora synthesizes vitamin K, affecting fracture risk.^[26]^ Supplementation with vitamin B12 and folic acid has been shown to reduce the risk of fracture, while gut microflora can synthesize B12 and thus influence the risk of fracture.^[27]^

More possible mechanisms require more basic experimental and clinical studies.

This study used the more comprehensive GWAS study on intestinal microorganisms and a MR study with a newer and more complete disease database with a sufficient sample size to enhance the study’s credibility. The Benjamini–Hochberg method was used to correct results at the phylum, family, and species levels to reduce the effect of Type II errors. The exposure and outcome of the study were both for people of European ancestry, and the 2 samples were relatively independent, which increased the reliability of the study results and eliminated the confounding effect of population differences.

This study also has the limitation that the taxonomic subtypes must be more complete to investigate the causal relationship between gut microbiota and fracture subtypes further. Second, the study population was of European origin, which may limit the applicability of the results of this study. Furthermore, the results of MR studies can only establish causal relationships between exposure and outcome but cannot further investigate the biological mechanisms of gut microflora and fracture. More observational and animal studies are needed to explore this further.

## 5. Conclusion

This study reveals a causal relationship between gut microflora and fracture risk and shows that the effect of flora abundance on fracture risk varies by flora taxa. The balance of gut microflora has value in reducing fracture risk. Further related studies can be refined based on the findings of this study so that the theoretical results can be translated into practice.

## Acknowledgments

The authors were thankful for all the researchers and participants who contributed to the MiBioGen and IEU Open GWAS consortiums.

## Author contributions

Writing—review and editing: Lingling Ju.

Data curation: Yong-kang Wei, Yanjun Liu.

Fromal analysis: Lingling Ju, Yong-kang Wei.

Writing—original draft: Lingling Ju, Yong-kang Wei.

Investigation: Yanjun Liu.
